# Transcriptomic Analysis of Resistant and Wild-Type Isolates Revealed Fludioxonil as a Candidate for Controlling the Emerging Isoprothiolane Resistant Populations of *Magnaporthe oryzae*

**DOI:** 10.3389/fmicb.2022.874497

**Published:** 2022-04-08

**Authors:** Zuo-Qian Wang, Fan-Zhu Meng, Liang-Fen Yin, Wei-Xiao Yin, Liang Lv, Xiao-Lin Yang, Xiang-Qian Chang, Shu Zhang, Chao-Xi Luo

**Affiliations:** ^1^Institute of Plant Protection and Soil Science, Hubei Academy of Agricultural Sciences, Wuhan, China; ^2^Key Laboratory of Integrated Pest Management on Crops in Central China, Ministry of Agriculture, Wuhan, China; ^3^Department of Plant Pathology, College of Plant Science and Technology, Huazhong Agricultural University, Wuhan, China; ^4^The Key Lab of Crop Disease Monitoring and Safety Control in Hubei Province, Huazhong Agricultural University, Wuhan, China

**Keywords:** isoprothiolane, Zn_2_Cys_6_ transcription factor, fludioxonil, resistance management, Hog1 MAPK pathway

## Abstract

The point mutation R343W in MoIRR, a putative Zn_2_Cys_6_ transcription factor, introduces isoprothiolane (IPT) resistance in *Magnaporthe oryzae*. However, the function of MoIRR has not been characterized. In this study, the function of MoIRR was investigated by subcellular localization observation, transcriptional autoactivation test, and transcriptomic analysis. As expected, GFP-tagged MoIRR was translocated in the nucleus, and its C-terminal could autonomously activate the expression of reporter genes *HIS3* and *α-galactosidase* in absence of any prey proteins in Y2HGold, suggesting that MoIRR was a typical transcription factor. Transcriptomic analysis was then performed for resistant mutant 1a_mut (R343W), knockout transformant ΔMoIRR-1, and their parental wild-type isolate H08-1a. Upregulated genes in both 1a_mut and ΔMoIRR-1 were involved in fungicide resistance-related KEGG pathways, including the glycerophospholipid metabolism and Hog1 MAPK pathways. All MoIRR deficiency-related IPT-resistant strains exhibited increased susceptibility to fludioxonil (FLU) that was due to the upregulation of Hog1 MAPK pathway genes. The results indicated a correlation between FLU susceptibility and MoIRR deficiency-related IPT resistance in *M. oryzae*. Thus, using a mixture of IPT and FLU could be a strategy to manage the IPT-resistant populations of *M. oryzae* in rice fields.

## Introduction

Rice blast caused by the ascomycete fungus, *Magnaporthe oryzae*, is one of the most threatening diseases in rice production (Wilson and Talbot, 2009). Fungicides have been developed to control this disease but few have remained effective after decades of use (Yamaguchi, 2004). In order to extend fungicide longevity and preserve the risk of resistance emerging, one fungicide was ideally applied in alternation or combination fungicides with different modes of action (Skamnioti and Gurr, 2009). Isoprothiolane (IPT), developed in the 1970s, was one of the most commonly used fungicides in developing Asia countries (Taninaka, 1980; Dung and Dung, 1999; Skamnioti and Gurr, 2009). IPT is known as a systemic fungicide with high protective and curative efficiency; it not only inhibits mycelial vegetable growth but also prevents infection pegs formation at a concentration of 10 μg/ml (Araki and Miyagi, 1976; Hirooka et al., 1982). Nevertheless, its mode of action has still not been fully clarified and IPT-resistant populations have been emerging in paddy fields (Zhang et al., 2013; Pan et al., 2021). Thus, unveiling its mode of action and elucidating resistance mechanisms are critical for effective management of resistant populations of *M. oryzae*.

IPT is mainly considered a choline biosynthesis inhibitor (CBI) because IPT treatment decreases choline transmethylation in mycelia (Yoshida et al., 1984). However, no variations in expression levels or gene coding sequences of choline synthesis genes were found between three laboratory-generated IPT-resistant mutants and their parental wild-type isolates in a previous study (Hu et al., 2014). In fact, mutations in a putative Zn_2_Cys_6_ transcription factor, MoIRR, were found to be responsible for IPT resistance. Those mutations were located in the Fungal_TF_MHR domain (fungal transcription factor middle homology region) which might affect the affinity between TFs and promoter due to conformation changes (Kolaczkowska et al., 2002). This correlation was determined by genetic transformation, i.e., mutated allele (R343W) was introduced into a sensitive isolate, and the subsequent transformants exhibited a similar level of resistance with resistant mutants (Wang et al., 2018).

Zn_2_Cys_6_ TFs are fungal exclusive zinc finger TFs that participate in life regulation, including vegetative growth, secondary metabolite production, meiosis, pathogenicity, stress response, and fungicide resistance (Small et al., 2001; Marui et al., 2002; Chooi et al., 2010; Schubert et al., 2011; Beaudoin et al., 2013; Lu et al., 2014). Most resistance-related TFs transcriptionally regulate transporters or fungicide target proteins. For instance, ABC transporter atrB expression is highly induced by Mrr1 mutagenesis, which confers multidrug resistance in *Botrytis cinerea* (Kretschmer et al., 2009). Cyp51A, the target of azoles fungicides, is also upregulated by AtrR in resistant isolates (Hagiwara et al., 2017). However, none of the MoIRR homologs have been characterized. Moreover, the genes regulated by MoIRR have not been well investigated either. Thus, a novel mechanism may exist for MoIRR involvement.

Typically, Zn_2_Cys_6_ TFs translocate into the nucleus, binding to specific DNA sequence at the promoter region and activating specific gene transcription (Vashee and Kodadek, 1995). Transcriptomic analysis of a deficient mutant provides a practicable strategy to explore the pathways regulated by the transcription factor (Taverner et al., 2004). This technique has also been widely used in the research on fungicide resistance; for instance, analysis of the transcriptomic data from resistant *Fusarium culmorum* isolates indicated that azole fungicide resistance mechanisms were related to a PDR transporter (Hellin et al., 2018). In this study, transcriptomic analysis was used to determine MoIRR mediated pathways and differentially expressed genes (DEGs).

The function of the putative Zn_2_Cys_6_ TF MoIRR has not been characterized, and the effect of MoIRR deficiency is unclear. Therefore, this study aimed to (i) characterize the localization and transcriptional activation of MoIRR, (ii) explore the regulatory network of MoIRR and effect of MoIRR deficiency, and (iii) develop scientific management strategy for controlling IPT-resistant populations in paddy fields.

## Materials and Methods

### Fungal Isolates and Growth Condition

Wild-type isolates, IPT-resistant mutants, MoIRR knockout transformants, and MoIRR-complemented transformants used in this study were collected or generated in previous studies (Table 1). Wild-type isolates H08-1a, H08-1c, and H08-6c were collected from infected rice leaves in a paddy field from Enshi, Hubei, China. The corresponding resistant mutants 1a_mut, 1c_mut, and 6c_mut were generated by chemical stimulation on IPT-containing PDA (Hu et al., 2014). MoIRR knockout transformants (ΔMoIRR-1, ΔMoIRR-2, and ΔMoIRR-3) were generated using a hygromycin-resistance split-marker approach that transformed split-marker fragments into protoplasts of the parental isolate H08-1a. MoIRR-complemented transformants (MoIRR-C-1, MoIRR-C-2, and MoIRR-C-3) were achieved by transformation of full-length *MoIRR* into ΔMoIRR-1 (Wang et al., 2018). MoIRR knockout transformants exhibited similar resistance levels against IPT compared with resistant mutants, and EC_50_ values of MoIRR-complemented transformants were recovered by expression of MoIRR (Table 1). All isolates were recovered from dried colonized filter paper disks stored at −20°C.

**Table 1:** List of isolates used in this study.

	Isolate	Genetic detail	IPT EC_50_ (μg/ml)	IPT sensitive	References
Parental wild-type	H08-1a	None	4.72	S	Hu et al., 2014
	H08-1c	None	4.1	S	Hu et al., 2014
	H08-6a	None	5.42	S	Hu et al., 2014
Resistant mutants^1^	1a_mut	R343W in MoIRR	19.23	R	Hu et al., 2014
	1c_mut	R345C in MoIRR	16.78	R	Hu et al., 2014
	6c_mut	Sixteen bp insertion at position 1,189 of MoIRR CDS	17.35	R	Hu et al., 2014
Knockout transformants^2^	ΔMoIRR-1	MoIRR was knocked out	17.93	R	Wang et al., 2018
	ΔMoIRR-2	MoIRR was knocked out	17.74	R	Wang et al., 2018
	ΔMoIRR-3	MoIRR was knocked out	17.38	R	Wang et al., 2018
MoIRR-complemented transformants^3^	MoIRR-C-1	MoIRR was complemented in ΔMoIRR-1	9.51	S	Wang et al., 2018
	MoIRR-C-2	MoIRR was complemented in ΔMoIRR-1	1.27	S	Wang et al., 2018
	MoIRR-C-3	MoIRR was complemented in ΔMoIRR-1	8.92	S	Wang et al., 2018

1 1a_mut, 1c_mut, and 6c_mut were generated by continuously exposing their parental isolates to IPT-amended PDA.

2 ΔMoIRR-1, -2, and -3 are the knockout transformants generated by transforming split-marker fragments into protoplasts of the parental isolate H08-1a.

3 MoIRR-C-1, -2, and -3 are the complemented transformants obtained by random insertion of fragment consisting of full-length MoIRR and its native promoter into the protoplasts of the knockout transformant ΔMoIRR-1.

### Subcellular Localization Observation

SLP-Local^1^ and WoLF PSORT^2^ were used to predict the subcellular localization of MoIRR based on an amino acid sequence similarity algorithm (Hua and Sun, 2001; Horton et al., 2007). The full-length (open reading frame and 3′ UTR) *MoIRR* gene was cloned with H08-1a gDNA and ligated into a pKNRG overexpression vector, containing a strong promoter and GFP tag at the 5′ end, using ClonExpress® II One Step Cloning Kit (Vazyme Biotechnol, Nanjing, China). The coding sequence of MoIRR was updated compared to the previous study, and the sequence was stored in GenBank with accession number OM311152. The plasmid was transformed into H08-1a protoplasts using PEG-mediated transformation. Transformants were screened with hygromycin B, further verified by PCR, and Quantitative real-time PCR (qRT-PCR). To observe subcellular localization, mycelia were cultured at 27°C for 3 days in a PDB medium with continuous shaking at 150 rpm, followed by observation using an inverted fluorescence microscope (*λ*_ex_ = 488 nm, *λ*_em_ = 503 nm; IX81, Olympus, Tokyo, Japan). Primers used in this study were listed in Supplementary Table S1.

### Transcriptional Activation Test

The transcriptional activity of MoIRR was determined using the Matchmaker® Gold Yeast Two-Hybrid System (Clontech, CA, United States). The sequence of MoIRR, MoIRR-N, and MoIRR-C was amplified using cDNA as templates and inserted into the plasmid pGBKT7 (BD). BD (negative control), BD-MoIRR, BD-MoIRR-N, and BD-MoIRR-C were transformed into yeast cells of strain Y2HGold separately, using LiAc-Mediated yeast transformation (Gietz and Schiestl, 2007). Primers are used to amplify fragments listed in Supplementary Table S1. The resulting yeast colonies were transferred to SD/-Trp medium, and tryptophan prototrophic mutants were selected and determined using specific PCR. A positive clone from each treatment was inoculated in SD/-Trp liquid medium and grown in shaking culture at 30°C for 48 h. When the OD_600_ reached around 0.4–0.5, the culture was centrifuged and the cell pellet was re-suspended in 0.9% NaCl solution. The yeast cell suspension was diluted to 1 × 10^7^, 2 × 10^6^, 5 × 10^5^, and 1 × 10^5^ cells per ml. They were inoculated on SD/-Trp-His medium and in addition presence of X-α-Gal (20 μl) using a drop method then incubated at 30°C for 3 days (Lou et al., 2015).

### Transcriptomic Analysis

The resistant mutant 1a_mut, MoIRR knockout transformant ΔMoIRR-1, and parental isolate H08-1a were inoculated into 100 ml flasks containing 40 ml PDB and shaking culture at 27°C, 150 rpm for 72 h. To ensure reliable gene expression profiles, each isolate had three biological replicates. Total RNA was extracted from the mycelia using Trizol reagent (Invitrogen, Carlsbad, CA, United States). Nine RNA samples were sent to Novogene Corporation (Novogene, Beijing, China) for RNA sequencing and basic analysis. RNA-Seq was performed on an Illumina HiSeq 4000 PE150 platform using 150 bp paired-end libraries with a 500-bp insertion. DEGs were calculated based on the normalized read count, and their corresponding values of *p* were determined. Pearson’s correlation coefficient was used to measure the expression profile similarities between samples. Differentially expressed genes were analyzed using the KEGG pathway database and enriched into KEGG pathways (Kanehisa et al., 2008). RNA-Seq reads data of H08-1a, ΔMoIRR-1, and 1a_mut were deposited at the GenBank SRA database under accession numbers SRX3362774, SRX3362767, SRX3362768, SRX3362771, SRX3362772, SRX3362773, SRX3362769, SRX3362770, and SRX3362775.

### Quantitative Real-Time PCR

Mycelia of H08-1a and ΔMoIRR-1 were cultured in 40 ml PDB and incubated at 27°C for 72 h with continuous shaking at 150 rpm. IPT-treatment samples were added 5 μg/ml IPT for 4 h before harvest, and mock samples were treated with the same volume of acetone that is the solvent of IPT. RNA was extracted using TRI Reagent (Ambion, Texas, United States). DNA residual in RNA was removed with DNase I (Thermo Fisher Scientific Inc., Vilnius, Lithuania). RNA was reverse transcribed into cDNA using a RevertAid First Strand cDNA Synthesis Kit with oligo (dT) 18 primers (Thermo Fisher Scientific Inc., Vilnius, Lithuania). The expression level of DEGs was evaluated using qRT-PCR on an Applied Biosystems StepOnePlus™ Real-Time System using SYBR® Select Master Mix (2×) (ABI, Massachusetts, United States) in 10 μl volumes. All the qRT-PCR experiments were performed with three independent biological repeats. The expression of genes was determined with primers listed in Supplementary Table S1. The expression of the DEGs was normalized to that of the β-tubulin gene, and relative gene expression was calculated using the comparative Ct (2^−ΔΔCt^) method (Wong and Medrano, 2005).

### Fungicide Sensitivity Assays

The sensitivity of MoIRR-deficient strains against FLU was assessed on fungicide-containing PDA with concentrations of 0, 0.3, 1, 3, 10, and 30 μg/ml. For each isolate, mycelial plugs of 5 mm diameter were taken from the edge of 5-day-old colonies grown on PDA and were placed at the center of FLU-containing PDA plates (9 cm diameter). After incubation at 27°C for 9 days, the colony diameters were measured from three replicate plates per concentration, per isolate. EC_50_ values (the fungicide concentration that inhibits mycelial growth by 50%) were obtained by regression analysis between growth inhibition rates and fungicide concentrations (Liang et al., 2015).

### Statistics

Statistical differences in the data were evaluated by one-way ANOVA with Duncan’s Multiple Range tests in SPSS for Windows Version 19.0 (SPSS Inc., Chicago, IL, United States).

## Results

### MoIRR Is Translocated Into the Nucleus and Has Transcriptional Activity

**Figure 1:**
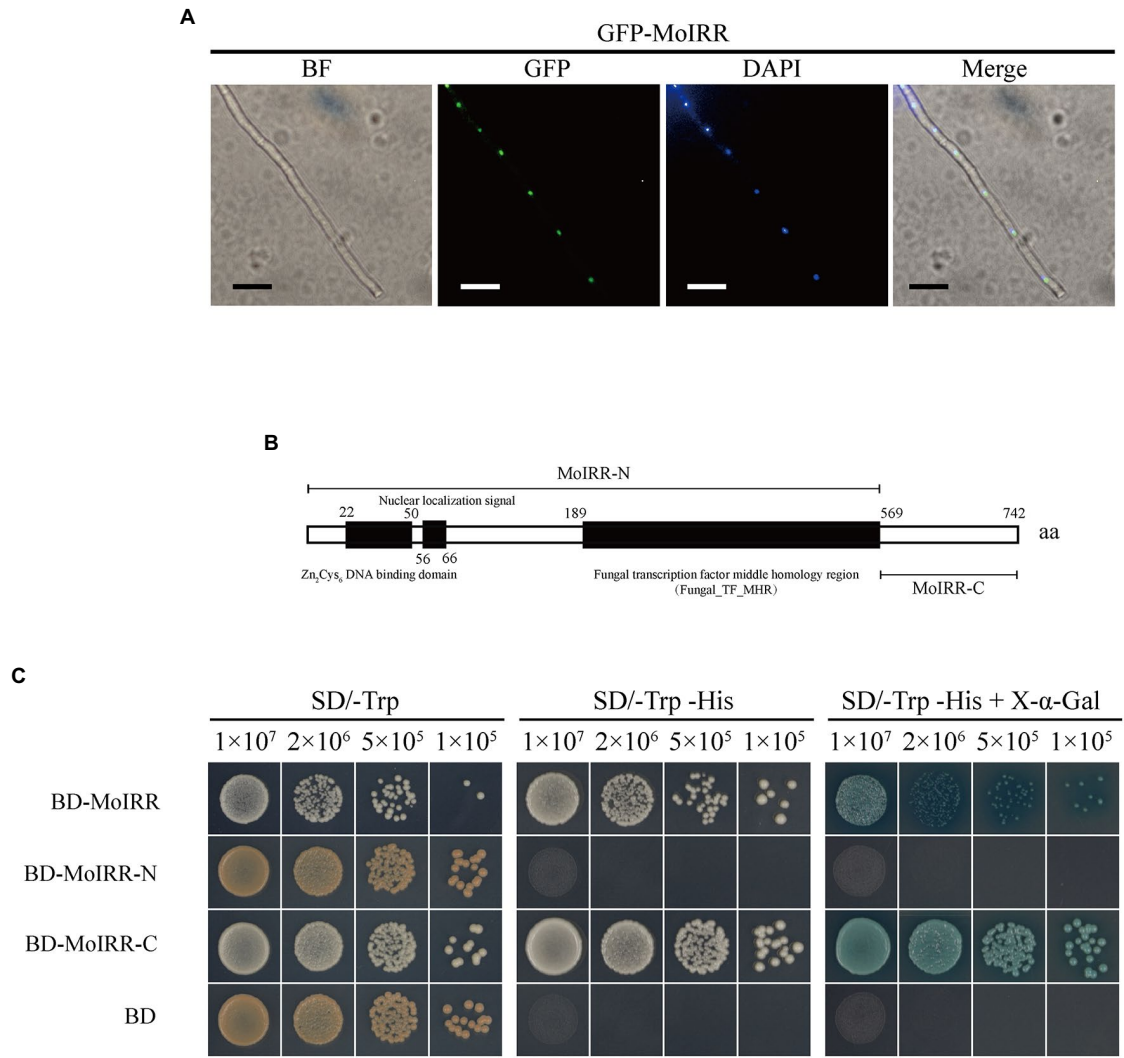
MoIRR is translocated into the nucleus and has transcriptional activity. **(A)** Subcellular localization of GFP-MoIRR fusion in the hyphae. The image of GFP showed the localization of MoIRR (second panels). Image of DAPI staining showed the localization of nuclear (third panels). The merged image of GFP and DAPI staining showed that the GFP-MoIRR fluorescence signal was overlapped with DAPI nuclear localization (fourth panel). Image of BF was captured under bright field (first panel). Isolates containing GFP-MoIRR were grown in a PDB liquid medium at 27°C for 2 days before observation. Images were captured using an inverted fluorescence microscope. Bars were 20 μm. **(B)** Schematic representation of *Magnaporthe oryzae* MoIRR. Prediction of Zn_2_Cys_6_ DNA binding domain, nuclear localization signal, and Fungal_TF_MHR domains was indicated by the black box. MoIRR-N and MoIRR-C fragments were indicated by a line segment. MoIRR, MoIRR-N, and MoIRR-C were cloned and inserted into vector pGBKT7. Then, plasmids were transformed into yeast cells for the transcriptional activation experiment. Domains indicted in the schematic were predicted with Conserved Domain Database in NCBI (https://www.ncbi.nlm.nih.gov/Structure/cdd/wrpsb.cgi). **(C)** Experimental determination of the transcriptional activity of MoIRR. Compared to BD, yeast transformants contained BD-MoIRR and BD-MoIRR-N could initiate strong gene transcription. The growth of yeast transformants on SD/-Trp (left panel, 30°C, 3 days) indicates complementation of the tryptophan auxotroph with the bait construct. Growth of the yeast transformants on SD/-Trp-His (middle panel, 30°C, 3 days) indicates activation of reporter gene *HIS3* transcription which could permit the cell to biosynthesize histidine and grow on His minimal medium, and appearance of blue color in the right panel indicates activation of α-galactosidase in the presence the chromogenic substrate X-a-Gal.

To investigate the localization of MoIRR, two pieces of subcellular localization predictive software were applied based on its amino acid sequence. Prediction results from SLP-Local indicated that MoIRR could be translocated into the nucleus or cytosol with a relatively low-reliability index (Supplementary Table S2). The result from WoLF PSORT II indicated ambiguous localization to the mitochondria, nucleus, or cytoplasm (Supplementary Table S3). Overall, translocation was mainly predicted into the nucleus, along with multiple destinations. To determine its real localization, GFP-tagged MoIRR was expressed in the wild-type isolate H08-1a, and GFP fluorescence signals were observed using a fluorescence microscope. As expected GFP signals were detected in the nucleus overlapped with the signal of 4,6-diamino-2-phenylindole (DAPI) staining, similar with the translocation pattern of typical transcription factors (Figure 1A; Supplementary Figure S1). Further, GAL4-based transcriptional activation test was used to determine whether the N-terminal or C-terminal of MoIRR could activate gene transcription (Clontech Laboratories, 2007). Full length, N-terminal, and C-terminal coding sequences of the gene were ligated into the PGBKT7 (BD) vector, respectively (Figure 1B). The results showed that indicated yeasts transformed with plasmid BD-MoIRR and BD-MoIRR-C could grow on SD/-Trp-His and blue colonies appear in the presence of X-α-Gal, indicating MoIRR and its C-terminal could autonomously activate transcription of reporter genes *HIS3* and *α-galactosidase*. However, transformants containing BD-MoIRR-N could not grow on SD/-Trp-His or SD/-Trp-His + X-α-Gal medium indicating MoIRR-N was lacking transcription activation domain. Results of transcriptional activation test were in agreement with that activation domain was localized at acidic C-terminal region in many cases of Zn2Cys6 transcription factors (Schjerling and Holmberg, 1996; Bovier et al., 2014). BD empty vector was transformed to be negative control (Figure 1C).

### Transcriptomic Analysis in Resistant Mutant 1a_mut and Knockout Transformant ΔMoIRR-1

To further identify the regulatory network of MoIRR and to elucidate the pathways involved in IPT resistance, transcriptomic analysis was performed in the resistant mutant 1a_mut, knockout transformant ΔMoIRR-1, and parental isolate H08-1a. Altogether, 1,218 upregulated genes and 1,126 downregulated genes were identified in 1a_mut. Further, there were 623 upregulated and 654 downregulated genes identified in ΔMoIRR-1.

**Figure 2:**
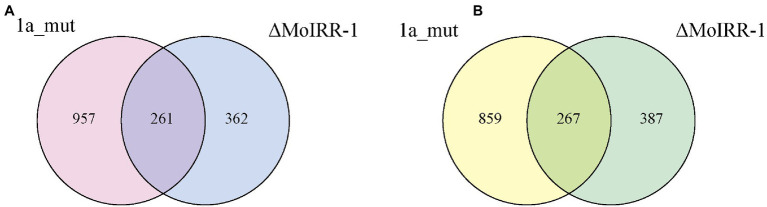
Venn diagrams showing the number of DEGs with similar expression pattern in transcriptomic analysis of 1a_mut and ΔMoIRR-1. **(A)** The number of unique and shared upregulated DEGs in 1a_mut and ΔMoIRR-1 compared to H08-1a. **(B)** The number of unique and shared downregulated DEGs in 1a_mut and ΔMoIRR-1 compared to H08-1a.

A Venn diagram was used to determine the genes that were simultaneously upregulated or downregulated in the two resistant strains, which were related to MoIRR deficiency. In total, 528 DEGs were sorted, and of these 261 were upregulated (Figure 2A) and 267 were downregulated in both 1a_mut and ΔMoIRR-1 (Figure 2B).

**Figure 3:**
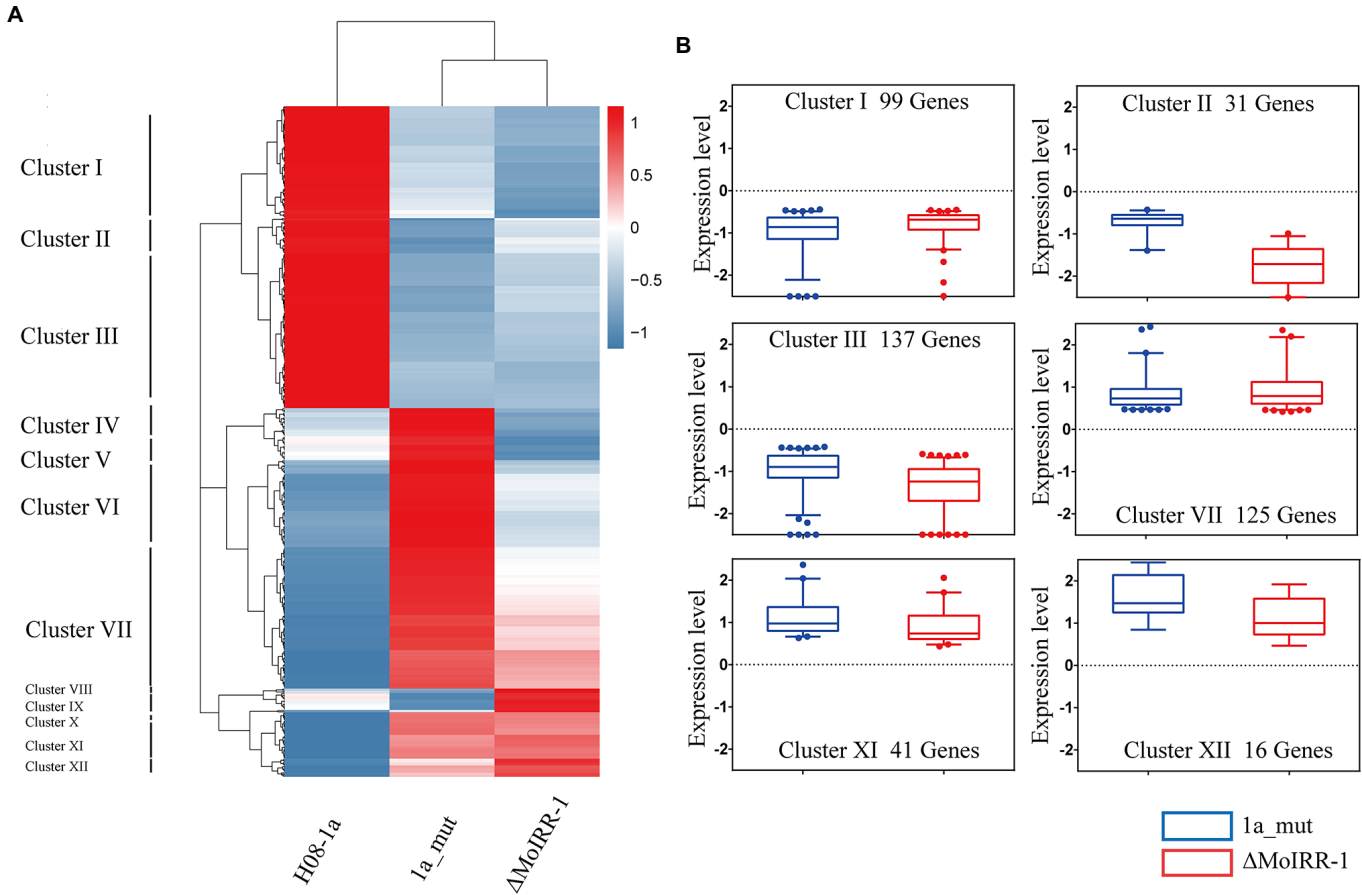
Hierarchical clustering showing expression patterns of DEGs in transcriptomic analysis of 1a_mut and ΔMoIRR-1. **(A)** Horizontal sample clustering showed that expression profiles of 1a_mut and ΔMoIRR-1 were closer than that of H08-1a. Vertical clustering of gene expression was divided into 12 clusters with multiple expression patterns. Color scale showing the level of gene expression of log_2_ (FPKM+1). **(B)** Box plot exhibiting the similar expression patterns of DEGs in six clusters between 1a_mut and ΔMoIRR-1. There were 9, 31, and 137 DEGs in Cluster I, Cluster II, and Cluster III were simultaneously downregulated in both 1a_mut and ΔMoIRR-1; 125, 41, and 16 DEGs in Cluster VII, Cluster XI, and Cluster XII were simultaneously upregulated in both 1a_mut and ΔMoIRR-1.

To elucidate the similarities and differences in the expression patterns among three isolates, a hierarchical clustering analysis was performed for simultaneously DEGs. The result of the sample clustering showed that 1a_mut and ΔMoIRR-1 belong to the same branch as indicated by their similar transcriptomic profile (Figure 3A). The expression profile of the DEGs could be divided into 12 clusters; we noticed that some genes exhibited similar expression patterns between 1a_mut and ΔMoIRR-1 in Clusters I, II, III, VII, XI, and XII. In clusters I, II, and III 99, 31, and 137 genes were downregulated in both 1a_mut and ΔMoIRR-1; in clusters VII, XI, and XII 125, 41, and 16 genes were upregulated in both 1a_mut and ΔMoIRR-1 (Figure 3B).

These potential MoIRR-regulated genes with similar expression patterns were then mapped to the KEGG pathways; of these, 32 were found to be involved in 13 pathways including the steroid biosynthesis pathway (mgr00100), oxidative phosphorylation pathway (mgr00190), five amino acid metabolism pathways (mgr00250, mgr00300, mgr00350, mgr360, and mgr00400), two lipid metabolism pathways (mgr00564 and mgr00600), three vitamin metabolism pathways (mgr00670, mgr00750, and mgr00760), and the Hog1 MAPK pathway (mgr04011; Supplementary Table S4). Notably, there were 11 genes in four pathways reported to be related to fungicide resistance, including three genes upregulated in the glycerophospholipid metabolism pathway (mgr00564), which were annotated as phospholipases that participated in phosphatidyl-choline biosynthesis. Further, demethylation inhibitor (DMI) fungicides target cytochrome P450 51 and two other steroid synthesis enzyme-encoding genes in the steroid biosynthesis pathway (mgr00100), which were found to be upregulated. Meanwhile, three genes in the oxidative phosphorylation pathway (mgr00190) were upregulated, among which MGG_00168 located at complex II (succinate dehydrogenase), is targeted by succinate dehydrogenase inhibitor (SDHI) fungicides (Veloukas et al., 2013). Lastly, Hog1 and Ypd1 (MGG_07173 and MGG_01822) in the Hog1 MAPK pathway (mgr00100) were also upregulated, knockout of Hog1 and Ypd1 reported leading to fludioxonil (FLU) resistance (Table 2; Jacob et al., 2015). Thus, the genes and pathways with similar expression patterns in both ΔMoIRR-1 and 1a_mut might be regulated by MoIRR.

**Table 2:** Potential fungicide resistance-related genes with similar expression patterns in the transcriptomic analysis of ΔMoIRR-1 and 1a_mut.

Gene ID	log_2_(Fold change)^1^				Gene function annotation
	1a_mut vs. H08_1a	Significance^2^	ΔMoIRR-1 vs. H08_1a	Significance	
Steroid biosynthesis (mgr00100)^3^					
MGG_06139	1.39	3.89E-6	1.23	8.65E-15	Squalene monooxygenase
MGG_04432	0.73	5.69E-5	1.26	1.35E-4	Cytochrome P450 51
MGG_06133	0.53	5.80E-4	0.65	8.26E-3	C4 methylsterol oxidase
Oxidative phosphorylation(mgr00190)					
MGG_00647	0.49	8.89E-5	0.74	4.19E-2	NADH dehydrogenase
MGG_04140	0.60	1.80E-4	0.74	3.82E-3	Mitochondrial NADH dehydrogenase
MGG_00168	0.54	6.17E-5	0.74	7.17E-3	Succinate dehydrogenase
Glycerophospholipid metabolism(mgr00564)					
MGG_05804	1.31	9.22E-3	0.60	5.15E-4	Phospholipase D
MGG_01523	1.13	3.30E-3	0.76	4.24E-6	Cytosolic phospholipase
Hog1 MAPK pathway(mgr04011)					
MGG_07173	0.53	3.62E-2	0.46	1.22E-2	Phosphorelay protein YPD1
MGG_01822	0.86	2.12E-3	0.62	2.59E-4	Hog1 MAP kinase

1 Differential gene expression was calculated based on normalized read count values and different expression levels are displayed in logarithmic form.

2 Adjusted values of *p* indicate the significance of differential expression.

3 KEGG pathway and their code number.

### Validation of MoIRR Deficiency-Related Genes Using qRT-PCR

**Figure 4:**
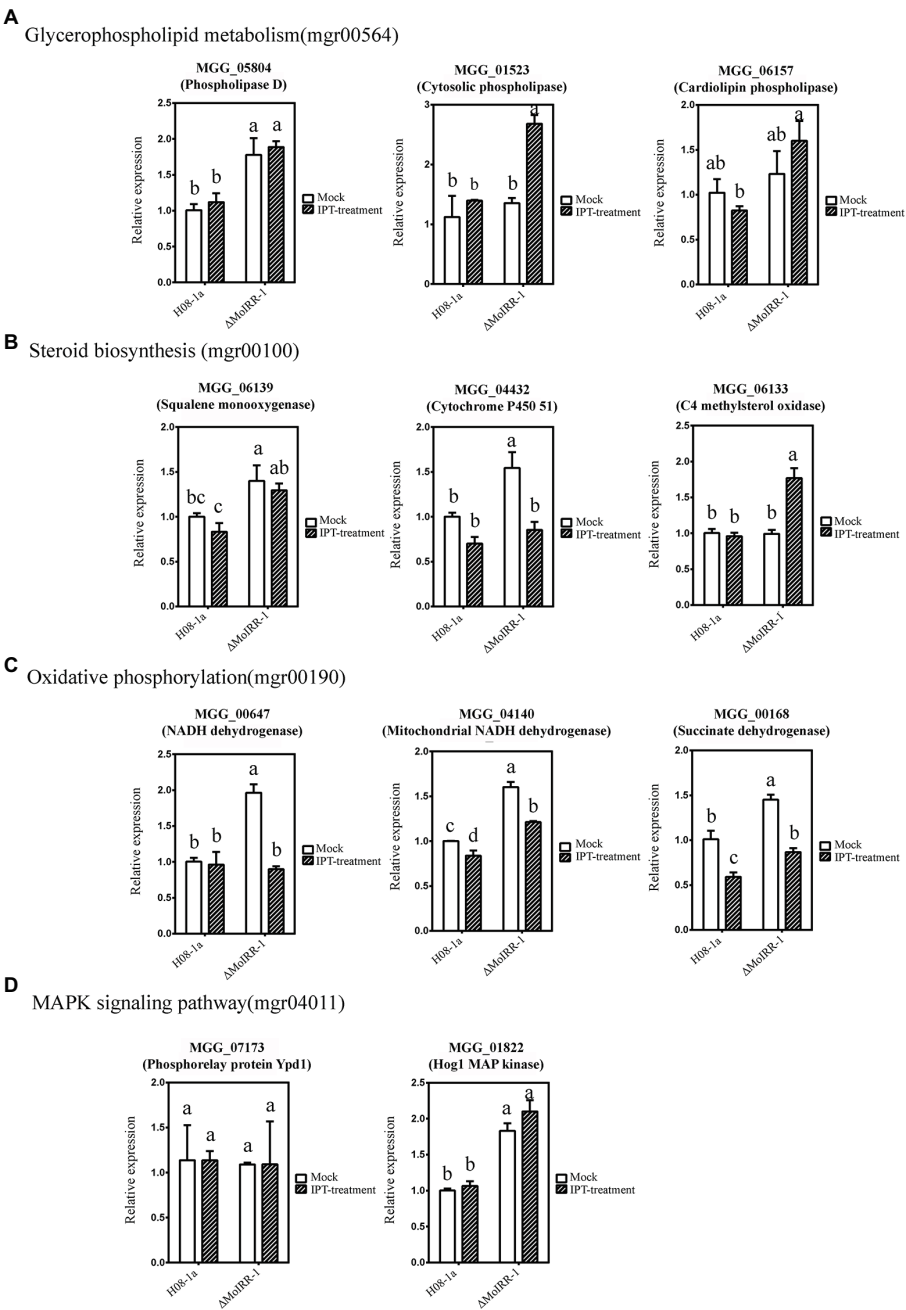
Validation of resistance-related gene expression by using quantitative real-time PCR (qRT-PCR). **(A)** Three genes were mapped in the glycerophospholipid metabolism pathway (mgr00564) reported being associated with IPT-inhibited phosphatidyl-choline biosynthesis. MGG_05804 annotated as phospholipase D was expressed significantly higher in ΔMoIRR-1 but did not induce by 5 μg/ml IPT treatment. Mycelia of H08-1a and ΔMoIRR-1 were inoculated into 40 ml PDB liquid medium separately and incubated at 27°C for 72 h with continuous shaking at 150 rpm. RNAs of mock samples were extracted from 4 h of acetone-treated cultures. RNAs of IPT-treatment samples were extracted from 4 h of 5 μg/ml IPT-treated cultures. Beta-tubulin was used as the reference gene in qRT-PCR. **(B)** Three genes were mapped in the steroid biosynthesis pathway (mgr00100) associated with DMI fungicide resistance. MGG_06139 annotated as squalene monooxygenase and MGG_04432 annotated as cytochrome P450 51 were expressed significantly higher in ΔMoIRR-1 but did not induce by 5 μg/ml IPT treatment. **(C)** Three genes were mapped in the oxidative phosphorylation pathway (mgr00190) associated with SDHI fungicide resistance. MGG_00647 annotated as NADH dehydrogenase, MGG_04140 annotated as mitochondrial NADH dehydrogenase, and MGG_00168 annotated as succinate dehydrogenase were expressed significantly higher in ΔMoIRR-1 but did not induce by 5 μg/ml IPT treatment. **(D)** Two genes were mapped in the Hog1 MAPK pathway (mgr04011), and their deletion was reported to confer resistance to FLU. MGG_01822 annotated as Hog1 MAP kinase was also expressed significantly higher in ΔMoIRR-1 but did not induce by 5 μg/ml IPT treatment. Different letters were calculated based on the replicates of treatments with Duncan test analysis indicate statistically significant differences (*p* = 0.05).

qRT-PCR was used to validate the expression levels of MoIRR deficiency-related genes such as phospholipase D (MGG_05804), cytosolic phospholipase (MGG_01523), and cardiolipin phospholipase (MGG_06157) in the glycerophospholipid metabolism pathway (Figure 4A); squalene monooxygenase (MGG_06139), cytochrome P450 51 (MGG_04432), and C4 methylsterol oxidase (MGG_06133) in the steroid biosynthesis pathway (Figure 4B); NADH dehydrogenase (MGG_00647), mitochondrial NADH dehydrogenase (MGG_04140), and succinate dehydrogenase (MGG_00168) in the oxidative phosphorylation pathway (Figure 4C); and Phosphorelay protein Ypd1 (MGG_07173) and Hog1 MAP kinase (MGG_01822) in the Hog1 MAPK pathway (Figure 4D). Among these, MGG_05804, MGG_06139, MGG_04432, MGG_00647, MGG_04140, MGG_00168, and MGG_01822 were expressed significantly higher in ΔMoIRR-1 than in wild-type H08-1a, consistent with results of transcriptomic analysis. However, none of them were upregulated under IPT-treatment, indicating that they were modulated by MoIRR but did not respond to IPT induction (Figure 4).

### All MoIRR-Deficient Strains Showed Increased Susceptibility to FLU

**Figure 5:**
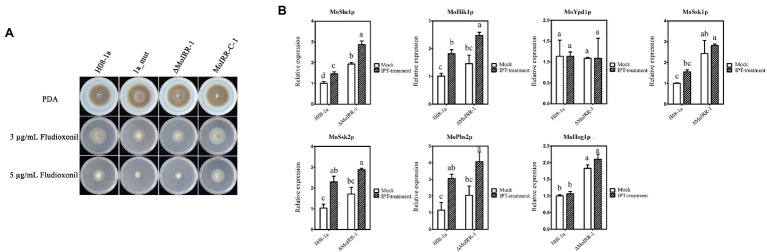
MoIRR-deficient strains showed increased susceptibility to FLU. **(A)** Growth inhibition of the resistant mutant 1a_mut, *MoIRR* knockout transformant ΔMoIRR-1, and MoIRR-complemented transformant MoIRR-C-1 compared to the wild-type isolate H08-1a on FLU-containing PDA. Growth of 1a_mut and ΔMoIRR-1 were inhibited more than H08-1a at both concentrations of 5 and 3 μg/ml FLU. But the growth of MoIRR-C-1 was similar to H08-1a. Mycelial plugs were taken from the edge of a 5 dpi colony of isolates or mutants and grown at 27°C for 6 days on PDA plates with FLU at a concentration of 0, 3, or 5 μg/ml. **(B)** The expression level of seven genes in Hog1 MAPK pathway was assessed using qRT-PCR in the ΔMoIRR-1 and H08-1a. MoSln1, MoSsk1, and MoHog1 were expressed higher in the ΔMoIRR-1 than in H08-1a. And expressions of MoSln1, MoHik1, MoSsk2, and MoPbs2 were induced under IPT treatment. RNAs extraction was the same with Figure 4. Different letters were calculated based on the replicates of treatments with Duncan test analysis indicate statistically significant differences (*p* = 0.05).

As mentioned above, we identified candidate genes that were upregulated in the knockout transformant ΔMoIRR-1. MGG_05804 is annotated to participate in phosphatidyl-choline biosynthesis. However, its overexpression transformants did not exhibit resistance against IPT (Supplementary Figure S2), suggesting that resistance might not be achieved by upregulation of phosphatidyl-choline biosynthesis genes. To investigate candidate genes in fungicide resistance-related pathways, 1a_mut and ΔMoIRR-1 were inoculated on DMI, SDHI, and phenylpyrrole fungicides containing PDA medium (Supplementary Figures S3, S4). Unexpectedly, both strains exhibited high susceptibility to FLU. To determine the correlation between the Hog1 MAPK pathway and MoIRR deficiency-related IPT resistance, FLU EC_50_ values of IPT-resistant mutants and MoIRR knockout transformants were tested according to mycelial growth on FLU-containing media. Remarkably, their growth inhibition rate was significantly increased compared to the wild type at concentrations of 3 and 5 μg/ml, while susceptibility decreased to wild-type level in MoIRR-C-1 that *MoIRR* was complemented into ΔMoIRR-1 (Figure 5A). Average EC_50_ values of wild type and MoIRR-complemented transformants were 3.76 ± 1.08 and 3.52 ± 0.43 μg/ml, while resistant mutants and knockout transformants were 1.04 ± 0.60 and 1.45 ± 0.43 μg/ml, respectively, reduced by 72.3% and 61.4% compared to wild-type isolates (Table 3). These results indicated that MoIRR deficiency-related IPT-resistant strains incidentally became more susceptible to FLU. To determine the reason they became more susceptible to FLU, the expression of seven genes (MoSln1, MoHik, MoYpd1, MoSsk1, MoSsk2, MoPbs2, and MoHog1) in the Hog1 MAPK pathway was evaluated by qRT-PCR in the knockout transformant ΔMoIRR-1 and wild-type H08-1a. The results showed that MoSln1, MoSsk1, and MoHog1 were expressed higher in the ΔMoIRR-1 than in H08-1a; further, expression of MoSln1, MoHik1, MoSsk2, and MoPbs2 was induced under IPT treatment (Figure 5B). These results indicated that the increased susceptibility to FLU was related to the upregulation of the genes in Hog1 MAPK pathway. Therefore, FLU could be a good candidate to control MoIRR deficiency-related IPT resistance in *M. oryzae*.

**Table 3:** Introduces isoprothiolane (IPT)-resistant mutants and MoIRR knockout transformants showed increased susceptibility to fludioxonil (FLU).

Isolate	FLU EC_50_ (μg/ml)^1^	IPT sensitivity
H08-1a	4.98	S
H08-1c	2.91	S
H08-6c	3.40	S
**Wild-type isolates**	3.76 ± 1.08a^2^	
1a_mut	1.11	R
1c_mut	1.00	R
6c_mut	1.01	R
**Resistant mutants**	1.04 ± 0.60b	
ΔMoIRR-1	0.96	R
ΔMoIRR-2	1.60	R
ΔMoIRR-3	1.78	R
**Knockout transformants**	1.45 ± 0.43b	
MoIRR-C-1	3.63	S
MoIRR-C-2	3.89	S
MoIRR-C-3	3.05	S
**MoIRR-complemented transformants**	3.52 ± 0.43a	

1 EC_50_ values of FLU were calculated based on relative mycelial growth regression analyses with concentrations of 0, 0.3, 1, 3, 10, and 30 μg/ml.

2 Mean ± SD (standard deviation of the mean). Different letters within a column were calculated based on the group of wild-type isolates, IPT-resistant mutants, MoIRR knockout transformants, and MoIRR-complemented transformants with Duncan test analysis indicate statistically significant differences (*p* = 0.05).

## Discussion

The involvement of Zn_2_Cys_6_ TFs in fungicide resistance has been described in several plants and human pathogenic fungi (Hellauer et al., 2002; Silver et al., 2004; Hagiwara et al., 2017). Mutations in a putative Zn_2_Cys_6_ transcription factor, MoIRR, were reported to confer resistance against IPT. MoIRR contains the GAL4-like Zn_2_Cys_6_ DNA binding domain and Fungal_TF_MHR domain (Wang et al., 2018). However, its transcriptional activation and regulation network have not been described.

Normally, fungicide resistance-related TFs are translocated into the nucleus and activate resistance gene transcription. In this study, MoIRR was confirmed to translocate into the nucleus and showed transactivation activity. Interestingly, transcriptional activation test revealed that only the C-terminal of MoIRR could transactivate reporter gene expression where none mutation was detected, suggesting IPT resistance-related mutations did not interrupt transcription activity of the gene but might only affect binding affinity with the promoter. Transcriptomic analysis is an effective way to investigate resistance mechanisms as well as to find the regulatory networks of TFs (Abdeen et al., 2010; Sang et al., 2015; Hellin et al., 2018; Zhang et al., 2020). Although heatmap clustering indicated that the transcriptomic profiles of resistant mutant 1a_mut and knockout transformant MoIRR-1 were closer than the wild type, some genes were expressed differently between them, suggesting that the mutation could not be simply equated with knockout. Normally, TFs regulated multiple genes by binding their TF binding sites (TFBS) with diverse affinity (Franco-Zorrilla and Solano, 2017). Therefore, even though 1a_mut acquired IPT resistance similar to ΔMoIRR-1, point mutations in the Fungal_TF_MHR may not affect regulating other non-resistance-related downstream genes.

Phosphatidyl-choline biosynthesis was previously studied as the target of IPT because its function was significantly decreased by IPT treatment in the mycelium (Uesugi, 2001). However, the relevant molecular evidence was lacking and the target protein was unknown. In this study, transcriptomic profiling showed that phosphatidyl-choline synthesis-related genes were expressed significantly higher in both 1a_mut and ΔMoIRR-1. However, none of their overexpression transformants acquired resistance against IPT. Although these results could not support the hypothesis that phosphatidyl-choline biosynthesis is the target of IPT, the correlation between phosphatidyl-choline biosynthesis- and IPT-resistant-related gene MoIRR was discovered, which may provide clue to explain mechanisms of resistance.

FLU is an effective phenylpyrrole fungicide that has been used against multiple leaf pathogens in both pre-harvest and post-harvest treatment. FLU mimics osmotic stress by activating the Hog1 MAPK pathway, resulting in mycelium cell death (Motoyama et al., 2005; Lew, 2010). Knockout transformants of these pathway components showed hypersensitivity to osmotic stress and exhibited resistance toward FLU (Jacob et al., 2015). Interestingly, IPT-resistant mutants and MoIRR knockout transformants both exhibited higher susceptibility to FLU in this study, suggesting a correlation between MoIRR deficiency and the Hog1 MAPK pathway. As expected, six genes in the Hog1 MAPK pathway were expressed higher in ΔMoIRR-1 than in the wild-type isolate H08-1a. Thus, the molecular mechanism of FLU susceptibility in MoIRR-deficient strains was unveiled and FLU could be used as a mixing partner for preventing IPT resistance occurrence and managing the emerged resistant populations of *M. oryzae*.

Fungicide resistance management has been a crucial problem since the 1970s, and the Fungicide Resistance Action Committee (FRAC) was thus established (Hollomon, 2015). The introduction of a mixture of fungicides is an effective tactic to control resistance development, and different mixing partners could show different performances in reducing resistance selection (van den Bosch et al., 2014). Usually, mixture of fungicides with different modes of action shows the better efficiency; for example, the efficiencies of penthiopyrad + cyproconazole and penthiopyrad + pyraclostrobin showed better performance than the fungicides (Culbreath et al., 2020). However, there has been no report about mixing fungicides selection based on the high-risk fungicide’s resistance mechanism. We first found that FLU could be a highly effective mixing partner for MoIRR deficiency-related IPT resistance management.

In conclusion, it was verified that MoIRR translocated to the nucleus and has transcription activity like a typical transcription factor. However, the binding of MoIRR to the target promoter could not be verified because the specific resistance genes were not found. Therefore, future work should identify promoter regions that interact with MoIRR using CHIP-seq. Further, a correlation between IPT resistance and the Hog1 MAPK pathway was discovered by comparing the transcriptomic profiles between the resistant mutant 1a_mut, knockout transformant ΔMoIRR-1, and the wild-type isolate H08-1a. All MoIRR-deficient strains exhibited increased susceptibility to FLU due to upregulation of the genes in the Hog1 MAPK pathway. Thus, a mixture of IPT and FLU should be considered to prevent IPT resistance occurrence and to manage the resistant populations of *M. oryzae* in paddy fields.

## Data Availability Statement

The datasets presented in this study can be found in online repositories. The names of the repository/repositories and accession number(s) can be found in the article/Supplementary Material.

## Author Contributions

C-XL and Z-QW conceived and designed the experiments. Z-QW, F-ZM, and L-FY performed the experiments. X-LY, W-XY, and X-QC analyzed the data. Z-QW and LL wrote the paper. SZ and C-XL supervised the study. All authors contributed to the article and approved the submitted version.

## Funding

This work is supported by the Natural Science Foundation of Hubei Province (2020CFB469), the Youth Science Foundation of Hubei Academy of Agricultural Sciences (2021NKYJJ07), and the National Key Research and Development Program (2016YFDO200807).

## Conflict of Interest

The authors declare that the research was conducted in the absence of any commercial or financial relationships that could be construed as a potential conflict of interest.

## Publisher’s Note

All claims expressed in this article are solely those of the authors and do not necessarily represent those of their affiliated organizations, or those of the publisher, the editors and the reviewers. Any product that may be evaluated in this article, or claim that may be made by its manufacturer, is not guaranteed or endorsed by the publisher.

## Footnotes

^1^ https://sunflower.kuicr.kyoto-u.ac.jp/~smatsuda/slplocal.html

^2^ https://www.genscript.com/wolf-psort.html
