# Supplementary material for: Transcriptomic Analysis of Resistant and Wild-Type Isolates Revealed Fludioxonil as a Candidate for Controlling the Emerging Isoprothiolane Resistant Populations of Magnaporthe oryzae
Source: Front Microbiol. 2022 Apr 8;13:874497. doi: 10.3389/fmicb.2022.874497 (PMC9024399; doi:10.3389/fmicb.2022.874497)
Supplement: Supplementary file 1 [file Data_Sheet_1.doc]

**Supplementary Table 1. Primers used in this study**

| **Primer pairs** | **Sequence（5'to 3'）** | **Description** |
| --- | --- | --- |
| pKGRG-MoIRR-F  pKGRG-MoIRR-R | TACATGGTAGATCTTATCGATAAGCTTATGTCGTCGGCCGCAGCGAG | Overexpression strain construction |
|  | GCGTTAATCTAGAACTAGTGGATCCCTATTGCCATGCTGATTGCG | Overexpression strain construction |
| BD-MoIRR-F | CATATGGCCATGGAGGCCATGTCGTCGGCCGCAGCGAG | pBDGAL4-MoIRR construction |
| BD-MoIRR-R | CAGGTCGACGGATCCCCGGCTATTGCCATGCTGATTGCG | pBDGAL4-MoIRR construction |
| BD-MoIRR-C-F | CATATGGCCATGGAGGCCatgCCATATCACCCAGAGTTACTCAA | pBDGAL4-MoIRR-C construction |
| BD-MoIRR-N-R | CAGGTCGACGGATCCCCGGctaTAATATATTCTCAACGGTCTCCCA | pBDGAL4-MoIRR-N construction |
| MGG_07312-F | GATGAAGCCAGGATTGATGTT | Expression detection |
| MGG_07312-R | CCCGTGTCCGTGTATGTAATA | Expression detection |
| MGG_11174-F | GTGAACGGAATGGCTATGAA | Expression detection |
| MGG_11174-R | CCCTTGACCTCTACCCTGAT | Expression detection |
| MGG_02897-F | AGACAGATAAAAGCGATGTTGAA | Expression detection |
| MGG_02897-R | CTAGATTTGCGGGTTGCAGT | Expression detection |
| MGG_00183-F | GAAGAGGGTCTCAAATCACTACA | Expression detection |
| MGG_00183-R | AACCAAGCAACATCAGCAAC | Expression detection |
| MGG_10268-F | GATGCGGAGAATGGCTGGAT | Expression detection |
| MGG_10268-R | CCGTAGTTTCCCTTGCCGAG | Expression detection |
| MGG_06139-F | GCGGACCACGGACAAGT | Expression detection |
| MGG_06139-R | GGGGTAATGGGCAGTCAA | Expression detection |
| MGG_04432-F | GGTGACCCCAACCACTTCCC | Expression detection |
| MGG_04432-R | GCCGTATCCGTAATCAATCTTC | Expression detection |
| MGG_06133-F | TGTGGATTGTTCTCCGTCTC | Expression detection |
| MGG_06133-R | CTTCCTTGCGGGCTCAT | Expression detection |
| MGG_00647-F | GTTGGACCCACGACACGA | Expression detection |
| MGG_00647-R | GCAAGAGGAGCCTGCGTAT | Expression detection |
| MGG_04140-F | ATCAGATATTCGTCGGGTGC | Expression detection |
| MGG_04140-R | GTCATAGTGGAGCAGGATGTAGT | Expression detection |
| MGG_00168-F  MGG_00168-R | CCTTGCGTCTTTCCATAACTT | Expression detection |
| MGG_04140-R | TGTCTTCTGCCTCTTTGCTC | Expression detection |
| MGG_05804-F  MGG_05804-R | ACACGGACATCTTCAGGCACC | Expression detection |
|  | CCGCCTCGCTAAGGAACTCA | Expression detection |
| MGG_01523-F  MGG_01523-R | CACAGGGCTATGGTAACACG | Expression detection |
|  | CACCCGACCCCGATTGA | Expression detection |
| MGG_06157-F  MGG_06157-R | AACTTTGAGCCCCTTACCC | Expression detection |
|  | TGTTCTTTGGCATACACCTTG | Expression detection |
| MGG_07173-F  MGG_07173-R | CTCGCAGTTCTCCCATCC | Expression detection |
|  | CGGCAACCGAATCACAA | Expression detection |
| MGG_01822-F  MGG_01822-R | GTCAAGTCACTCCCCAAGCG | Expression detection |
|  | TCCACAGGTAGGTCTGCGTC | Expression detection |
| beta-tubulin-qRTp1-F  beta-tubulin-qRTp1-R | GTGACCCTCGCAACGGAAAG | Reference gene |
|  | CGACGAACTGGATGCTACGC | Reference gene |
|  |  |  |

**Supplementary Table 2. Sub-cellular localization predicted with SLP-Local**

| **Sequence label** | **Predicted location** | **Score** | **RI^1^** |
| --- | --- | --- | --- |
| MoIRR | Nucleus or cytosol | 0.94 | 2 |

^1^RI is a Reliability Index ranging from 1 to 10. This indicates prediction reliability. As the value of RI becomes larger, the prediction result becomes more reliable.

**Supplementary Table 3. Sub-cellular localization** predicted with WoLF PSORT II

| **Prediction sites** | **Count^1^** |
| --- | --- |
| Mitochondria | 12 |
| Nucleus | 7 |
| Cytoplasm | 6 |
| others | 2 |

^1^The number of signal sequence matched sites.

**Supplementary Table 4. Genes with similar expression patterns in both ΔMoIRR-1 and 1a_mut.**

| **Gene Name** | **log^2^FoldChange^1^**  **(ΔMoIRR-1)** | **P-value^2^** | **significant**  **(ΔMoIRR-1)** | **log^2^FoldChange**  **(1a_mut)** | | **P-value** | **Significant**  **(1a_mut)** | **KO^3^** | **function annotation** |
| --- | --- | --- | --- | --- | --- | --- | --- | --- | --- |
| **Steroid biosynthesis (mgr00100)** | | |  | |  |  |  |  |  |
| MGG_06139 | 1.232 | 3.89E-06 | UP | | 1.3853 | 8.65E-15 | UP | K00511 | squalene monooxygenase |
| MGG_04432 | 1.2641 | 5.69E-05 | UP | | 0.73235 | 0.000135 | UP | K05917 | cytochrome P450 51 |
| MGG_06133 | 0.65187 | 0.000581 | UP | | 0.52691 | 0.008264 | UP | K07750 | C-4 methylsterol oxidase |
| **Oxidative phosphorylation (mgr00190)** | | | | |  |  |  |  |  |
| MGG_00647 | 0.73591 | 8.9E-05 | UP | | 0.48979 | 0.041943 | UP | K03942 | hypothetical protein |
| MGG_04140 | 0.73568 | 0.000179 | UP | | 0.60084 | 0.00382 | UP | K03885 | mitochondrial NADH dehydrogenase |
| MGG_00168 | 0.73808 | 6.17E-05 | UP | | 0.54447 | 0.007174 | UP | K00234 | hypothetical protein |
| **Alanine, aspartate,e and glutamate metabolism (mgr00250)** | | | | | | |  |  |  |
| MGG_08450 | 1.6967 | 1.55E-12 | UP | | 1.5033 | 5.22E-14 | UP | K01953 | asparagine synthase |
| MGG_04156 | 0.55573 | 0.004386 | UP | | 0.42087 | 0.046135 | UP | K14454 | aspartate aminotransferase |
| MGG_06530 | 0.48556 | 0.019586 | UP | | 0.59843 | 0.002886 | UP | K14455 | aspartate aminotransferase |
| MGG_07187 | 0.99121 | 0.002671 | UP | | 1.0657 | 4.99E-09 | UP | K00264 | glutamate synthase |
| **Lysine biosynthesis (mgr00300)** | | |  | |  |  |  |  |  |
| MGG_04842 | 0.44852 | 0.049313 | UP | | 0.83204 | 0.002186 | UP | K01705 | Homoaconitase |
| MGG_09919 | 0.54321 | 0.009053 | UP | | 1.9924 | 2.8E-28 | UP | K00838 | amino transferase |
| MGG_06157 | -0.75839 | 0.000154 | DOWN | | -1.1423 | 7.34E-09 | DOWN | K14454 | alpha/beta hydrolase |
| **Tyrosine metabolism (mgr00350)** | | |  | |  |  |  |  |  |
| MGG_08455 | 0.59831 | 0.010867 | UP | | 0.86964 | 0.000447 | UP | K00505 | hypothetical protein |
| MGG_06530 | 0.48556 | 0.019586 | UP | | 0.59843 | 0.002886 | UP | K14455 | aspartate aminotransferase |
| MGG_04156 | 0.55573 | 0.004386 | UP | | 0.42087 | 0.046135 | UP | K14454 | aspartate aminotransferase |
| **Phenylalanine metabolism (mgr360)** | | |  | |  |  |  |  |  |
| MGG_06530 | 0.48556 | 0.019586 | UP | | 0.59843 | 0.002886 | UP | K14455 | aspartate aminotransferase |
| MGG_09919 | 0.54321 | 0.009053 | UP | | 1.9924 | 2.8E-28 | UP | K00838 | amino transferase |
| MGG_04156 | 0.55573 | 0.004386 | UP | | 0.42087 | 0.046135 | UP | K14454 | aspartate aminotransferase |
| MGG_03462 | 0.56292 | 0.035175 | UP | | 1.357 | 1.61E-12 | UP | K01426 | hypothetical protein |
| **Phenylalanine, tyros,ine, and tryptophan biosynthesis (mgr00400)** | | | | | | | |  |  |
| MGG_06530 | 0.48556 | 0.019586 | UP | | 0.59843 | 0.002886 | UP | K14455 | aspartate aminotransferase |
| MGG_09919 | 0.54321 | 0.009053 | UP | | 1.9924 | 2.8E-28 | UP | K00838 | amino transferase |
| MGG_04156 | 0.55573 | 0.004386 | UP | | 0.42087 | 0.046135 | UP | K14454 | aspartate aminotransferase |
| **Glycerophospholipid metabolism (mgr00564)** | | | | |  |  |  |  |  |
| MGG_05804 | 0.60493 | 0.009224 | UP | | 1.3141 | 0.000515 | UP | K01115 | phospholipase Dp1 |
| MGG_01523 | 0.75645 | 0.003301 | UP | | 1.1256 | 4.24E-06 | UP | K16342 | cytosolic phospholipase A2 |
| **Sphingolipid metabolism (mgr00600)** | | |  | |  |  |  |  |  |
| MGG_06250 | 0.58484 | 0.004509 | UP | | 0.5425 | 0.013377 | UP | K04713 | hypothetical protein |
| **One carbon pool by folate (mgr00670)** | | | | |  |  |  |  |  |
| MGG_04435 | 0.43105 | 0.048785 | UP | | 0.75449 | 7.92E-05 | UP | K00602 | purine biosynthesis protein |
| MGG_01728 | 0.62669 | 0.003621 | UP | | 1.1615 | 8.02E-06 | UP | K00297 | methylenetetrahydrofolate reductase 1 |
| **Vitamin B6 metabolism (mgr00750)** | | |  | |  |  |  |  |  |
| MGG_01535 | 0.56437 | 0.004275 | UP | | 0.8748 | 2.32E-06 | UP | K00275 | pyridoxamine 5'-phosphate oxidase |
| **Nicotinate and nicotinamid metabolism (mgr00760)** | | | | | |  |  |  |  |
| MGG_07539 | 0.7529 | 7.31E-05 | UP | | 0.66424 | 0.000992 | UP | K00323 | NAD(P) transhydrogenase |
| **Hog1 MAPK pathway (mgr04011)** | | |  | |  |  |  |  |  |
| MGG_07173 | 0.46497 | 0.036236 | UP | | 0.53357 | 0.012221 | UP | K11232 | hypothetical protein |
| MGG_01822 | 0.61868 | 0.002121 | UP | | 0.86336 | 0.000259 | UP | K04441 | CMGC/MAPK/P38 protein kinase |

^1^Gene different expressions were calculated based on normalized readcount values and different expression levels displayed in logarithmic form.

^2^*P*-value was post adjusted.

^3^KEGG pathway KO number


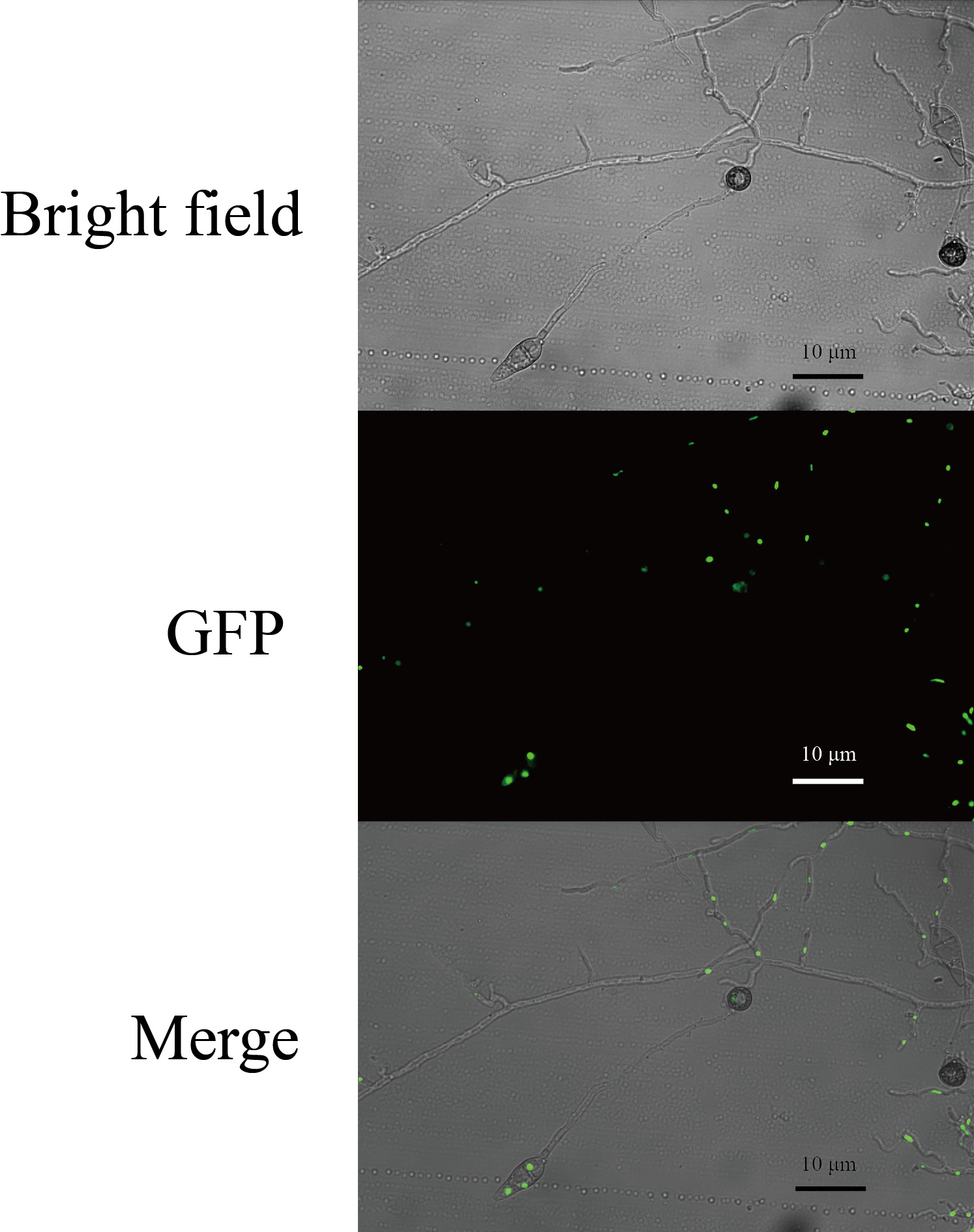


**Fig. S1. Fluorescent signal of GFP-MoIRR was observed located in nuclear during differentiation processes germination.**


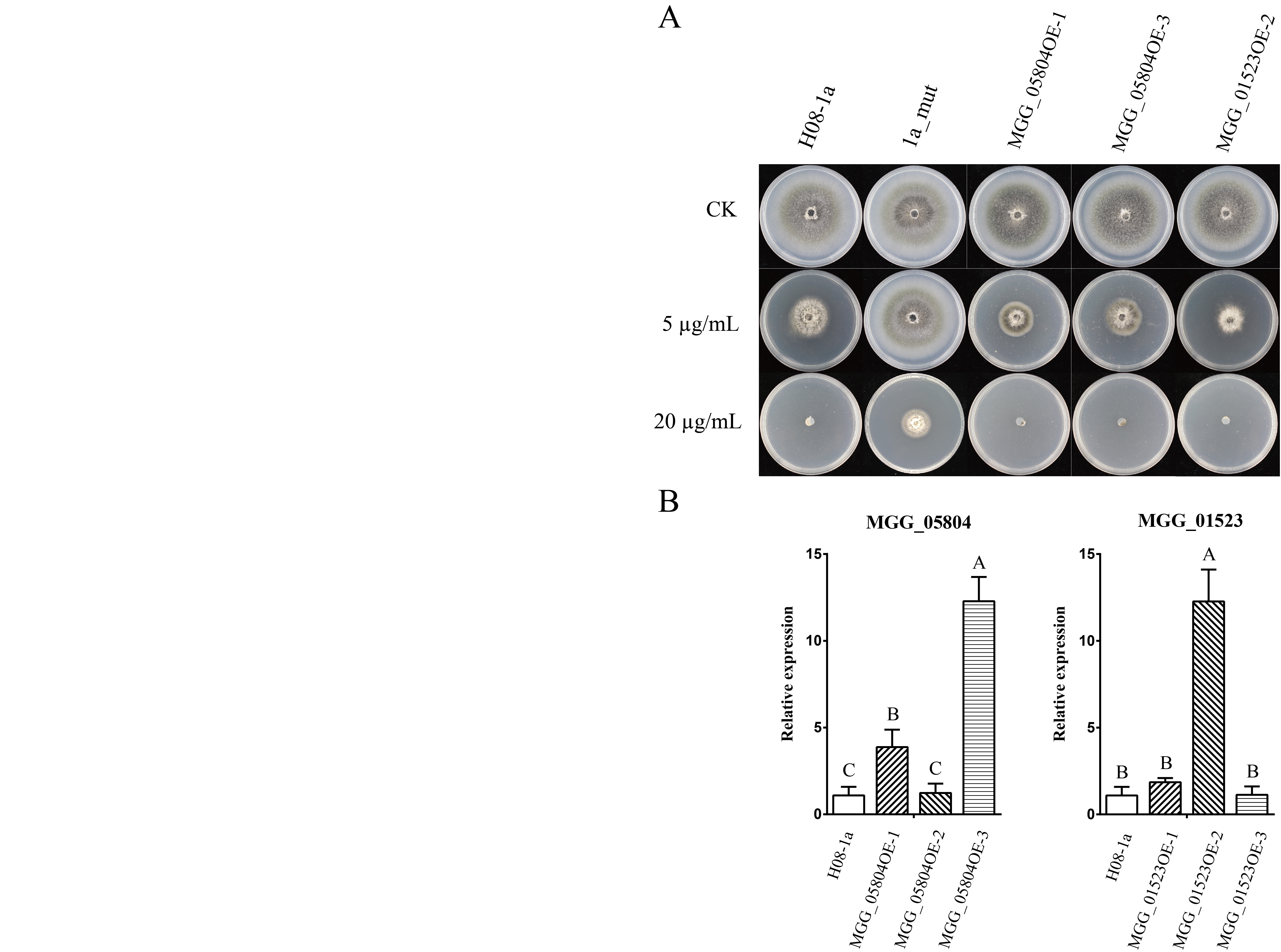


**Fig. S2. Overexpression transformants of two phosphatidyl-choline biosynthesis-related DEGs did not exhibit resistance against IPT**

A), Inhibition of overexpression transformants at IPT amended PDA media.

B), Expression levels of overexpression transformants were verified by qRT-PCR.


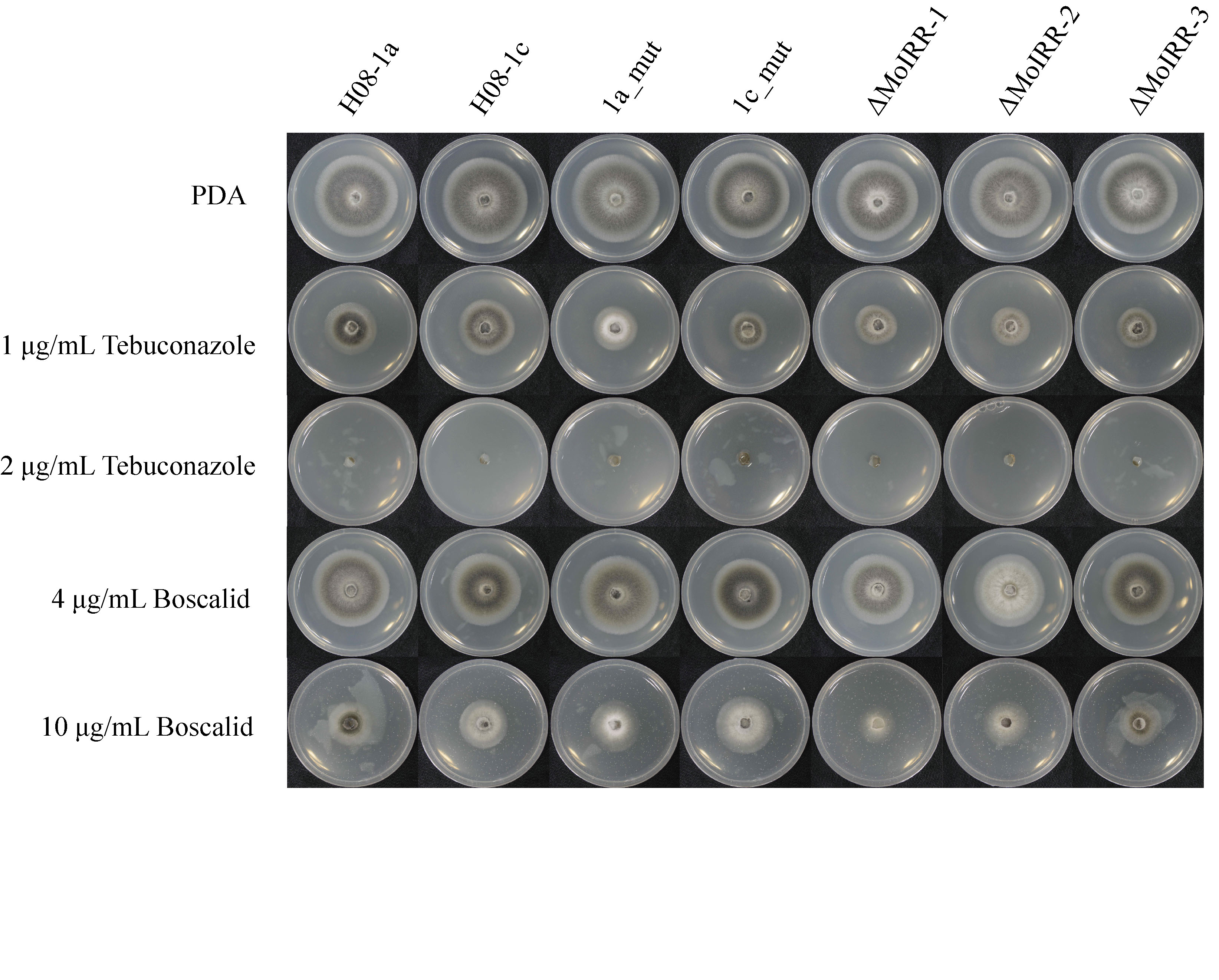


**Fig. S3. Growth of two resistant mutants, their parental wild-type isolates and three knockout transformants were assayed on different concentrations of tebuconazole and boscalid amended media (PDA, 27°C, 5d)**

**
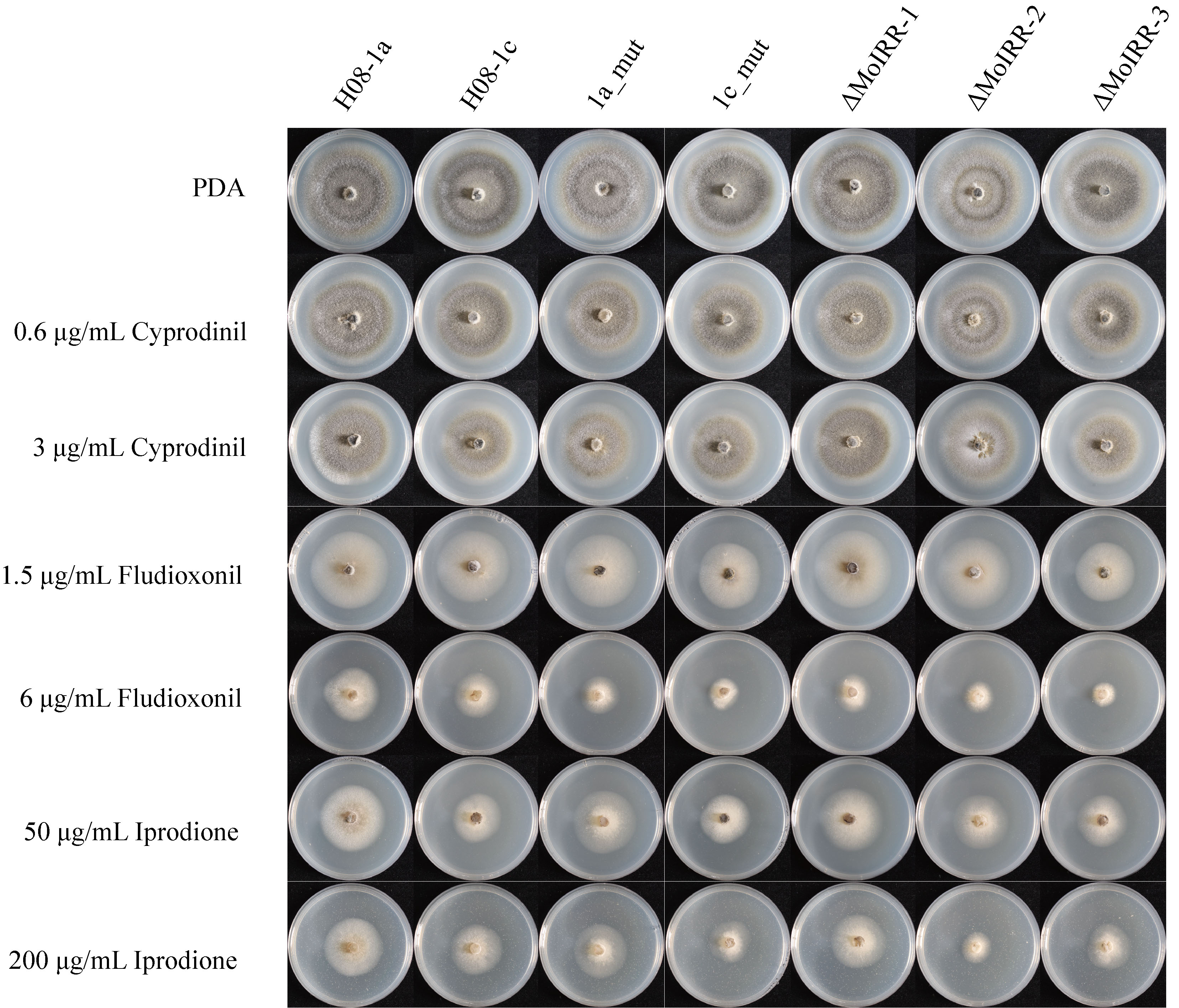
**

**Fig. S4. Growth of two resistant mutants, their parental wild-type isolates and three knockout transformants were assayed on different concentrations of cyprodinil, fludioxonil, iprodione (PDA, 27°C, 7d)**
